# Cell-of-origin of diffuse large B-cell lymphomas determined by the Lymph2Cx assay: better prognostic indicator than Hans algorithm

**DOI:** 10.18632/oncotarget.15782

**Published:** 2017-02-28

**Authors:** Nara Yoon, Soomin Ahn, Hae Yong Yoo, Suk Jin Kim, Won Seog Kim, Young Hyeh Ko

**Affiliations:** ^1^ Department of Pathology, The Catholic University of Korea Incheon St. Mary's Hospital, Incheon, Korea; ^2^ Department of Patholgy, Ewha Womans University Medical Center, Ewha Womans University School of Medicine, Seoul, Korea; ^3^ Department of Health Sciences and Technology, Samsung Advanced Institute for Health Sciences and Technology, Sungkyunkwan University, Seoul, Korea; ^4^ Division of Hematology and Oncology, Department of Medicine, Samsung Medical Center, Sungkyunkwan University School of Medicine, Seoul, Korea; ^5^ Department of Pathology and Translational Genomics, Samsung Medical Center, Sungkyunkwan University School of Medicine, Seoul, Korea

**Keywords:** lymphoma, large B-cell, diffuse, gene expression profiling, Lymph2CX

## Abstract

Diffuse large B-cell lymphomas (DLBCLs) are clinically heterogeneous and need a biomarker that can predict the outcome of treatments accurately. To assess the prognostic significance of the cell-of-origin type for DLBCLs, we applied the Lymph2Cx assay using a NanoString gene expression platform on formalin-fixed paraffin wax-embedded pretreatment tissues obtained from 82 patients with *de novo* DLBCL, not otherwise specified. All patients were treated with rituximab plus cyclophosphamide, doxorubicin, vincristine, and prednisone (R-CHOP) as the first line of chemotherapy. Based on the expression levels of Bcl-6, CD10, and MUM-1 measured by immunohistochemistry, cases were subdivided into germinal center B-cell (GCB) and non-GCB types according to the Hans algorithm. NanoString assay was performed on 82 cases. The Lymph2Cx assay successfully classified 82 cases into three categories: activated B-cell (ABC), GCB, and unclassified types. The concordance rate between the Lymph2Cx assay and the Hans algorithm was 73.6%. The Lymph2Cx-defined ABC type had significantly poorer outcomes compared with the GCB type (5-year overall survival, GCB vs. ABC, 96.6% vs. 77.1%, *P* = 0.020; 5-year disease-free survival, GCB vs. ABC, 96.6% vs. 79.2%, *P* = 0.018). In contrast, no significant differences were observed in survival between the two patient subgroups with DLBCL types classified by the Hans algorithm. The Lymph2Cx assay is a robust, reliable method for predicting the outcome of patients with DLBCL treated with R-CHOP chemotherapy.

## INTRODUCTION

Diffuse large B-cell lymphoma (DLBCL) is the most frequent type of non-Hodgkin's malignant lymphoma comprising 30–40% of adult lymphomas [1] and is a clinically heterogeneous disease showing unpredictable outcomes [2–5]. The most powerful prognostic factor is the International Prognostic Index (IPI) based on clinical and biochemical parameters including age, Eastern Cooperative Oncology Group (ECOG) performance status, tumor stage, extranodal involvement, and lactate dehydrogenase (LDH) level [4, 6]. In 2000, Alizadeh *et al*. used “Lymphochip” cDNA microarrays and found that the “Cell-of-Origin” (COO) of DLBCL could be divided into activated B-cell (ABC) and germinal center B-cell (GCB) types, with significant prognostic values [7]. The Leukemia Lymphoma Molecular Profiling Project applied the same microarray gene expression profiling (GEP) to an expanded cohort and identified three subgroups within DLBCL: ABC, GCB, and unclassified subgroups. This suggested that a GEP-defined COO might serve as an independent prognostic biomarker [8]. The GCB subtype appears to arise from germinal-center B cells and is associated with two molecular events: recurrent gene (14:18) translocations involving *BCL-2* and *C-REL* amplification [2, 8–10]. The ABC subtype might arise from a post-germinal center B cell and has frequent amplifications of the oncogene *SPIB*, recurrent trisomy for chromosome 3, and activation of the antiapoptotic nuclear factor (NF)-κB signaling pathway [2, 8–11].

The microarray-based GEP technique using RNA extracted from frozen tissue was the original standard for determining the COO type of DLBCL [10, 12]. With limited access to frozen samples, immunohistochemistry (IHC)-based classifications have been developed during the past decade [13–16]. Among them, the Hans algorithm, a classification based on three antibodies (CD10, BCL6, and MUM1) is widely used in place of microarrays [12, 15]. However, its inherent subjectivity and variability in scoring, lowers the reliability of such IHC-based methodology [17–21].

The nCounter platform of NanoString Technologies (Seattle, WA, USA) is useful for the direct multiplex measurement of gene expression using formalin-fixed paraffin wax-embedded (FFPE) tissues, and various clinical research studies using this platform have been performed [22–30]. Scott *et al*. [31] have recently published a 20-gene version of a NanoString code set for a COO typing assay of DLBCL named Lymph2Cx. Fifteen genes, along with five housekeeping genes, were selected among 93 genes, which were identified based on their ability to accurately replicate the COO model of Lenz *et al*. [9, 31] Here, we applied the Lymph2Cx NanoString nCounter gene expression system to FFPE pretreatment tissue samples from 82 patients with DLBCL and evaluated its predictive value compared with the Hans algorithm.

## RESULTS

### Patient population

The study included 51 (62.2%) male and 31 (37.8%) female patients, with a mean age of 60 years (range 18–82). Of these, 73 (89%) patients had a good performance status (ECOG grade 0 or 1). More than two-thirds (70.7%) had extranodal lymphomas and the rest (29.3%) had nodal lymphomas (Table 1). The extranodal sites included in our study are the following: stomach, colon, small intestine, appendix, breast, spleen, testis, brain, head and neck, thyroid, kidney, soft tissue, ovary, bone, and heart. As for the distribution of IPI populations, forty nine (59.8%) cases were categorized as low IPI risk group (0-1), 15 (18.3%) cases as low Intermediate (2), 10 (12.2%) cases as high intermediate (3), 8 (9.8%) cases as high score (4-5). Follow-up data were available for all cases with a mean follow-up of 50 months (range 0–145).

**Table 1:** Associations between clinical features and COO types determined by the Hans algorithm and Lymph2Cx assay

Characteristics	Total(n = 82)	COO assay by Hans algorithm, n (%)			COO assay by Lymph2Cx, *n* (%)			
		GCB(n = 39)	Non-GCB(n = 43)	P	GCB(n = 29)	ABC(n = 48)	Unclassified (n = 5)	P
Gender:				0.734				0.977
Male	51 (62.2)	25 (64.1)	26 (60.5)		19 (65.5)	28 (58.3)	4 (80.0)	
Female	31 (37.8)	14 (35.9)	17 (39.5)		10 (34.5)	20 (41.7)	1 (20.0)	
Primary site:				0.776				0.682
Nodal	24 (29.3)	12 (30.8)	12 (27.9)		8 (27.6)	16 (33.3)	0 (0)	
Extranodal	58 (70.7)	27 (69.2)	31 (72.1)		21 (72.4)	32 (66.7)	5 (100)	
Age, years:				0.002*				0.016*
≤ 60	40 (48.8)	26 (66.7)	14 (32.6)		20 (69.0)	18 (37.5)	2 (40.0)	
> 60	42 (51.2)	13 (33.3)	29 (67.4)		9 (31.0)	30 (62.5)	3 (60.0)	
ECOG performance status:				0.613				0.823
< 2	73 (89.0)	34 (87.2)	39 (90.7)		26 (89.7)	42 (87.5)	5 (100)	
≥ 2	9 (11.0)	5 (12.8)	4 (9.3)		3 (10.3)	6 (12.5)	0 (0)	
Serum LDH:				0.882				0.647
Normal	54 (65.9)	26 (66.7)	28 (65.1)		21 (72.4)	30 (62.5)	3 (60.0)	
Elevated	28 (34.1)	13 (33.3)	15 (34.9)		8 (27.6)	18 (37.5)	2 (40.0)	
Stage:				0.750				0.630
< 3	54 (65.9)	25 (64.1)	29 (63.4)		21 (72.4)	29 (60.4)	4 (80.0)	
≥ 3	28 (34.1)	14 (35.9)	14 (32.6)		8 (27.6)	19 (39.6)	1 (20.0)	
Extranodal involvement:				0.841				0.286
< 2	68 (82.9)	32 (82.1)	36 (83.7)		26 (89.7)	38 (79.2)	4 (80.0)	
≥ 2	14 (17.1)	7 (17.9)	7 (16.3)		3 (10.3)	10 (20.8)	1 (20.0)	
IPI grade:				0.869				0.566
Low (0–1)	49 (59.8)	22 (56.4)	27 (62.8)		18 (62.1)	27 (56.3)	4 (80.0)	
Intermediate (2–3)	25 (30.5)	14 (35.9)	11 (25.6)		10 (34.5)	15 (31.3)	0 (0)	
High (4-5)	8 (9.8)	3 (7.7)	5 (11.6)		1 (3.4)	6 (12.5)	1 (20.0)	

COO, cell of origin; ABC, activated B-like cell; GCB, germinal center B-like; ECOG, Eastern Cooperative Oncology Group performance; LDH, lactate dehydrogenase; IPI, international prognostic index.

### COO assay by Lymph2Cx

This cohort consisted of 43 non-GCB and 39 GCB DLBCLs, as classified by the Hans algorithm. All FFPE samples yielded sufficient RNA for the NanoString technology analyses. The results of the COO assay by Lymph2Cx are summarized in Table 2. Of the 82 cases, 48 (58.5%) were classified as ABC; 29 (35.4%) as GCB, and five (6.1%) were unclassified by Lymph2Cx. Of 43 non-GCB cases classified by the Hans algorithm, 36 (83.7%) were classified as ABC, three (7.0%) as GCB, and four (9.3%) were unclassified by the Lymph2Cx assay. In 39 GCB cases classified by the Hans algorithm, 12 (30.8%) were classified as ABC, 26 (66.7%) as GCB, and one (2.6%) was unclassified by the Lympn2Cx assay. The overall concordance rate between the COO determined by the Hans algorithm and the Lymph2Cx assay was 73.6%, 80.5% when unclassified cases were removed

**Table 2:** Comparison between the Hans algorithm and Lymph2Cx assay

		COO by Lymph2Cx, n (%)			
		ABC (n = 48)	GCB (n = 29)	Unclassified (n = 5)	P
COO by Hans algorithm; *n* (%)	Non-GCB (n = 43)	36 (75.0)	3 (10.3)	4 (80.0)	< 0.001
	GCB (n = 39)	12 (25.0)	26 (89.7)	1 (20.0)	

COO, cell of origin; ABC, activated B-like cell; GCB, germinal center B-like.

### Clinical characteristics of COO groups classified by the Lymph2Cx assay or the Hans algorithm

Clinical correlations with COO subgroups classified by the Lymph2Cx assay or the Hans algorithm are shown in Table 1. Compared with the GCB type, patients were significantly older in those classified as having a non-GCB type by the Hans algorithm (*P* = 0.002), and having an ABC type or unclassified by the Lymph2Cx subtype (*P* = 0.016). Gender, primary sites, ECOG performance scores, serum LDH levels, Ann Arbor tumor stage, extranodal site number, and IPI showed no associations.

### Univariate survival analysis

**Figure 1:**
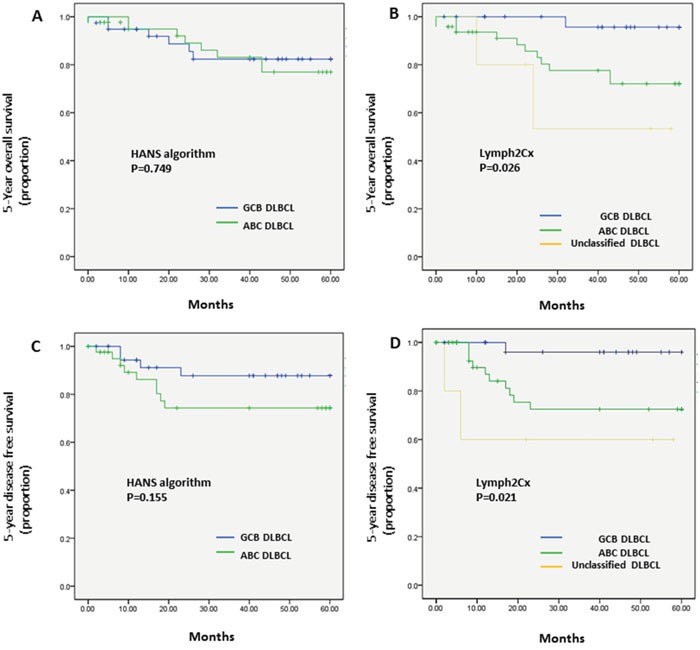
Kaplan–Meier analysis of 5-year OS and DFS in the patients with DLBCL types classified by the Hans algorithm A., C. or the Lymph2Cx assay B., D (A) COO assay by the Hans algorithm showed no difference in 5-year OS (*P* = 0.749) between GCB and non-GCB types. (B) Lymph2Cx-defined GCB type gave the most favorable outcome among three Lymph2Cx-defined subgroups (GCB vs. ABC vs. unclassified; 96.6% vs. 77.1% vs. 60%, respectively; *P* = 0.026). (C) COO assay by the Hans algorithm showed no difference in the 5-year DFS rate (*P* = 0.155) between the GCB and non-GCB types. (D) The Lymph2Cx-defined GCB type had the best patient survival among the three subgroups (GCB vs. ABC vs. unclassified; 96.6% vs. 79.2% vs. 60%, respectively; *P* = 0.021).

Kaplan–Meier plots of OS and DFS showed distinct differences in the 5-year patient survival rates between COO types classified using the Lymph2Cx system. Those with the GCB type had an improved 5-year OS (GCB vs. ABC vs. unclassified: 96.6% vs. 77.1% vs. 60%, respectively; *P* = 0.026) and 5-year DFS (GCB vs. ABC vs. unclassified: 96.6% vs. 79.2% vs. 60%, respectively; *P* = 0.021) compared with the ABC and unclassified types categorized by the Lymph2Cx method (Figure 1). Patients with the unclassified type of tumor had the worst OS and DFS rates. Patients with the COO type determined by the Hans algorithm showed no significant differences in 5-year OS (*P* = 0.749) or 5-year DFS rates (*P* = 0.155) between the GCB and non-GCB groups (Figure 1).

When the unclassified groups were excluded from the Lymph2Cx data, the differences in 5-year OS (GCB vs. ABC, 96.6% vs. 77.1%; *P* = 0.020) (Supplementary Figure 1) and 5-year DFS (GCB vs. ABC, 96.6% vs. 79.2%; *P* = 0.018) (Supplementary Figure 2) remained significant.

### Multivariate survival analysis

IPI scores 0–1 vs. 2–5 and the COO type determined by the Lymph2Cx assay were combined in a multivariate Cox Proportional Hazards model; this revealed that both were independent predictors of 5-year OS and DFS rates (Table 3).

**Table 3:** Multivariate survival analyses with combined IPI scores (0–1 vs. 2–5) and the COO determined by the Lymph2Cx assay

Variable	Unfavorable category	5-year OS			5-year DFS		
		HR	95% CI	P	HR	95% CI	P
COO by Lymph2Cx				0.024*			0.029*
	ABC	7.840	1.011–60.784	0.049	7.654	0.979–59.873	0.052
	Unclassified	33.669	2.700–419.908	0.006	30.449	2.454–377.770	0.008
IPI score	Intermediate/High	5.119	1.423–18.417	0.012*	3.518	1.010–12.253	0.048*

COO, Cell of origin; IPI, international prognostic index; OS, overall survival; DFS, disease free survival; HR, hazard ratio; CI, confidence interval.

## DISCUSSION

The development of DNA microarray techniques has provided effective tools for exploring the molecular features of DLBCL and has provided knowledge of specific genes associated with particular responses to chemotherapy [7, 8, 10, 31–34]. Although microarray GEP approaches are standard methods for determining the COO type of DLBCL [7, 8, 10], they are expensive and have poor flexibility and reproducibility when evaluating low-quality RNA samples, especially from FFPE samples; moreover, they still require RNA extraction from frozen tissues for high quality data [22, 24–26, 32]. For this reason, despite its high accuracy, microarray-based COO profiling is not applicable in clinical practices in which FFPE samples are used widely.

IHC-based methodology is rapid, cost-effective and readily available, so it has been widely developed and adopted in clinical practice and research [14–16, 33]. The Hans algorithm subdivides cases into GCB or non-GCB types by combining the results for the CD10, BCL-6, and MUM1 antibodies [15]. This algorithm showed 80% concordance with the GEP classification and similar patient survival outcomes [15]. The Choi algorithm adding FOXP1 and GCET1 to Hans algorithm also demonstrated high concordance (93%) with a GEP classification [12]. A Tally method substituting BCL6 for the LMO2 antibody showed a better ability to predict COO than other IHC-based algorithms [16]. The three algorithms were recently compared with GEP in 108 biopsy samples, and the study showed that the Hans and Choi algorithms were significantly more predictive of OS and progression-free survival (PFS) than the Tally method [34]. However, the value of IHC methods for assessing COO has become questionable because of poor concordance with GEP methods, inferior accuracy and reproducibility, and a lack of prognostic utility [4, 11, 15–19]. Accordingly, there is a need for developing reproducible, reliable and clinically applicable techniques for assessing the COO types in patients with DLBCL.

The NanoString technology uses digitally colored code sets that are attached to sequence-specific probes. By direct measurement of mRNA, it offers highly sensitive, reproducible and fully quantitative results on FFPE and frozen tissue samples [22, 23, 32, 35–37]. The technique covers a large number of genes, enables complex genomic analysis including the detection of gene fusions, and requires a low RNA input of as little as 200 ng. A previous study demonstrated a strong concordance between patient-matched frozen and FFPE material, showing the applicability of the NanoString platform to FFPE samples [22]. Scott *et al*. [31] selected 20 of the most predictive genes of a NanoString code set for the COO assay and generated a predictive model named Lymph2Cx, and the scores produced by two independent laboratories showed a very high concordance.

In this study, we applied the Lymph2Cx assay to 82 FFPE pretreatment samples from patients with DLBCLs who were treated with R-CHOP and compared it with the Hans algorithm. The correlation between the Hans algorithm and the Lymph2Cx assay was poor, with a discordancy rate of 26.4%. Of cases classified as ABC by the Lymph2Cx assay, 25% were misclassified as GCB by the Hans algorithm and 3% of cases classified as GCB by the lymph2Cx assay were misclassified as non-GCB by the Hans algorithm. We re-reviewed IHC slides of 12 GCB cases by Hans reclassified as ABC by Lymph2Cx. Eleven of 12 cases showed diffuse positivity for CD10 in more than 75% of tumor cells without background overstaining. One case with CD10 negativity was diffusely positive for BCL6 and negative for MUM1. Regarding BCL-6, 12 cases were positive with more than 60%. MUM1 was also diffusely positive for 11 cases without background stain. Therefore, misclassification based on the Hans algorithm does not seem to be caused by technical error of IHC

The Hans algorithm has been reported to have a misclassification rate of 19.7% when compared with GEP data; this might arise from technical factors related to staining, interpretation, and scoring of the data [18, 38]. However, Barrans *et al*. pointed out that decision trees of IHC algorithms use sequential rather than parallel consideration of markers, inherently fail to capture the overall pattern of gene expression, and lead to a lack of clinical correlation [17].

It is well established that patients with the GCB type of COO show significantly better OS than those with the ABC type by GEP classification [7, 8, 20, 33, 39]. Our COO typing assay made using the Lymph2Cx system, maintained the same prognostic significance as previous reports [9, 31, 35]. The Lymph2Cx-defined ABC type patient groups had significantly worse outcome than those with the GCB type whereas the COO types assigned by the Hans algorithm did not show significantly different outcomes in this cohort.

Distinct from the binary IHC groups, a third type of COO has been recognized in GEP studies in which the tumor cannot be assigned to either GCB or ABC groups [8]. This biologically distinct subgroup was named “type 3” and is now known as “unclassified” [8, 40]. In this study, five (6.1 %) cases labeled as the unclassified type had the worst outcome among all three groups as determined by the Lymph2Cx assay. This was inconsistent with previous Lymph2Cx results, in which the unclassified group of patients was shown to have an intermediate prognosis between the GCB and ABC types [31, 35]. Of five unclassified DLBCLs, two patients had recurrence and died of tumor progression. The poor survival for this type might have an association with underlying infectious diseases that compromised the patient's condition leading to cancer progression. One patient (#1) had a history of tuberculosis (TB) and this had been reactivated at the time of death. The second patient (#3) was a carrier of the Hepatitis B virus (HBV) and had synchronous early gastric cancer (Table 4). The study of Barrans *et al*. [17] presented the best survival of patients with unclassified DLBCLs (designated as Type III in their study), but this differed from a previous GEP study [8]. They speculated that this disagreement arose from the different mix of cases in their cohort showing that a number of different entities were likely to be included in the Type III group, including DLBCL of T-cell rich type and DLBCL presenting in extranodal sites [17]. Their data along with ours suggest that unclassified types of DLBCL might have variable clinical outcomes because of the heterogeneity of study populations. This phenomenon was emphasized by a relatively smaller proportion of the unclassified group, comprising about 5–15% of DLBCLs [31, 40].

**Table 4:** Immunohistochemical and clinical findings of unclassified DLBCL types determined by the Lymph2Cx assay

Case No.	CD10 (%)	BCL6 (%)	MUM1 (%)	Han's algorithm	Recurrence	Death from DLBCL progression	Clinicopathological history
1	0	5	95	ABC	Yes	Yes	Reactivated TB
2	0	30	70	ABC	No	No	
3	0	40	70	ABC	Yes	Yes	Synchronous EGC (submucosal); HBV carrier
4	0	90	40	ABC	No	No	C-MYC FISH+
5	90	95	70	GCB	No	No	

TB, tuberculosis; EGC, early gastric cancer; HBV, hepatitis B virus.

The limitation of our study is that the patient population is not representative of typical nodal DLBCL. We selected mainly surgical specimens rather than biopsy tissues for evaluation of whole protein expression pattern by immunohistochemistry. Therefore, extra-nodal sites were overly selected. In addition, there are some differences in patient population between ours and previous studies [31, 35]. Compared to Scott et al's series, this study had more low-stage disease, more low-IPI score, a lower proportion of GCB cases, and better OS rates [31, 35]

### Conclusion

In this study, the Lymph2Cx assay could divide DLBCLs into three molecular subgroups with distinctive prognoses, whereas the IHC-based Hans algorithm failed to reproduce this. Thus, the Lymph2Cx assay could predict survival independent of the IPI score and was successfully applied to FFPE tissue samples.

## MATERIALS AND METHODS

We retrospectively evaluated pretreatment samples taken from 100 patients with DLBCL who underwent excisional biopsy including resection of solid organs before chemotherapy in Samsung Medical Center between 2004 and 2015. Representative FFPE tissues of the 100 cases were selected from the archived histopathology files of Samsung Medical Center. A hematoxylin and eosin-stained section from each sample was assessed to confirm the diagnoses and tumor content. After experiments, patients who were not treated with rituximab plus cyclophosphamide, doxorubicin, vincristine, and prednisone (R-CHOP) or those with post-transplant lymphoproliferative and Epstein–Barr virus (EBV)-positive DLBCL tumors were excluded, as well as one patient who died of diabetic renal disease, by reviewing electronic medical records. Finally, 82 cases of DLBCL, not otherwise specified (NOS) remained for data analysis.

### Ethics statement

The study was approved by the institutional review board of the Samsung Medical Center (IRB NO:2011-11-056).

### CD10, BLC-6, and MUM-1 detection with IHC

IHC was performed for CD10, BCL6, and MUM1 antibodies at the time of the initial diagnosis. FFPE tissues were cut into 4 μm sections and then stained using an automated system (Technomate 1000, DakoCytomation, DAKO, Glostrup, Denmark) using a standard method. The samples were analyzed semi-quantitatively by two pathologists (N. Yoon and Y. Ko). Based on the Hans algorithm, CD10, BCL6, and MUM1 expression levels were evaluated with 30% cutoff values for the proportions of tumor cells stained. Consensus on discordant cases was reached by a joint review on a multi-head microscope.

### NanoString-based multigene assay

NanoString assay was performed according to literature published [31]. The probe sequences, the procedures, an aliquot of the oligonucleotide standards were kindly provided by Prof. Rimsza L. at Mayo clinic. Total RNA was extracted from two 4-μm thick sections from FFPE tumor tissues using High Pure RNA Paraffin kits (Roche Diagnostic, Mannheim, Germany) after removing non-tumorous elements by manual macrodissection guided by consulting the hematoxylin and eosin-stained slides. Nucleic acids were extracted using Qiagen AllPrep FFPE kits (Qiagen, Hildon, Germany), and digital GEP was performed on 200 ng aliquots of RNA using NanoString technology. The Lympho2Cx code set (Nanostring Technologies, Seattle, WA, USA) was used for gene expression analyses. The data were normalized to the mean expression levels of internal reference genes with cut off value 20. Standard QC was employed by nSolver™ Analysis Software (NanoString Technologies, WA) with flagging of any sample with a total of the positive spike-in controls being outside of 0.3 to 3 times the geometric mean of the total positive spike-in for that cartridge [31]. Data processing and the determination of COO type were done through the websitehttps://llmpp.nih.gov/LYMPHCX/index.shtml.

### Statistical analysis

Statistical analysis was performed using SPSS statistical software (v. 20.0; IBM Corp., Armonk, NY, USA). Clinicopathological associations with COO types derived by the Lymph2Cx assay and the Hans algorithm were analyzed using chi-squared tests and linear-by-linear association. Overall survival (OS) and disease-free survival (DFS) rates of the patients with particular COO types were estimated using Kaplan–Meier and multivariate Cox Proportional Hazards analyses. A *p*-value < 0.05 was considered significant.

## SUPPLEMENTARY MATERIALS FIGURES


