# Supplementary material for: Cell-of-origin of diffuse large B-cell lymphomas determined by the Lymph2Cx assay: better prognostic indicator than Hans algorithm
Source: Oncotarget. 2017 Feb 28;8(13):22014–22. doi: 10.18632/oncotarget.15782 (PMC5400642; doi:10.18632/oncotarget.15782)
Supplement: Supplementary file 1 [file oncotarget-08-22014-s001.pdf]

Cell-of-origin of diffuse large B-cell lymphomas determined by the Lymph2Cx assay: better prognostic indicator than Hans algorithm

SUPPLEMENTARY FIGURES

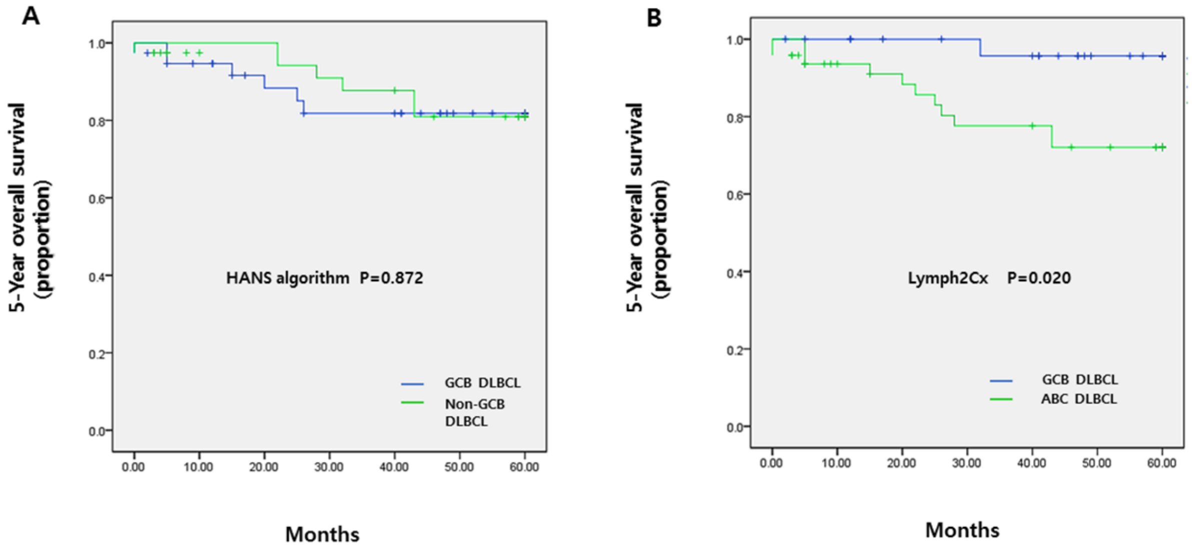

Supplementary Figure 1: Kaplan–Meier analyses of 5-year OS in the patients with DLBCL types classified by the Hans algorithm A. or the Lymph2Cx assay B. excluding unclassified groups by the Lymph2Cx assay. (A) Patients with the COO type determined by the Hans algorithm showed no difference in 5-year OS (P = 0.872) between GCB and non-GCB types. (B) Patients with the Lymph2Cx-defined GCB type had significantly better OS than those with the ABC type (GCB vs. ABC; 96.6% vs. 77.1%, P = 0.020).

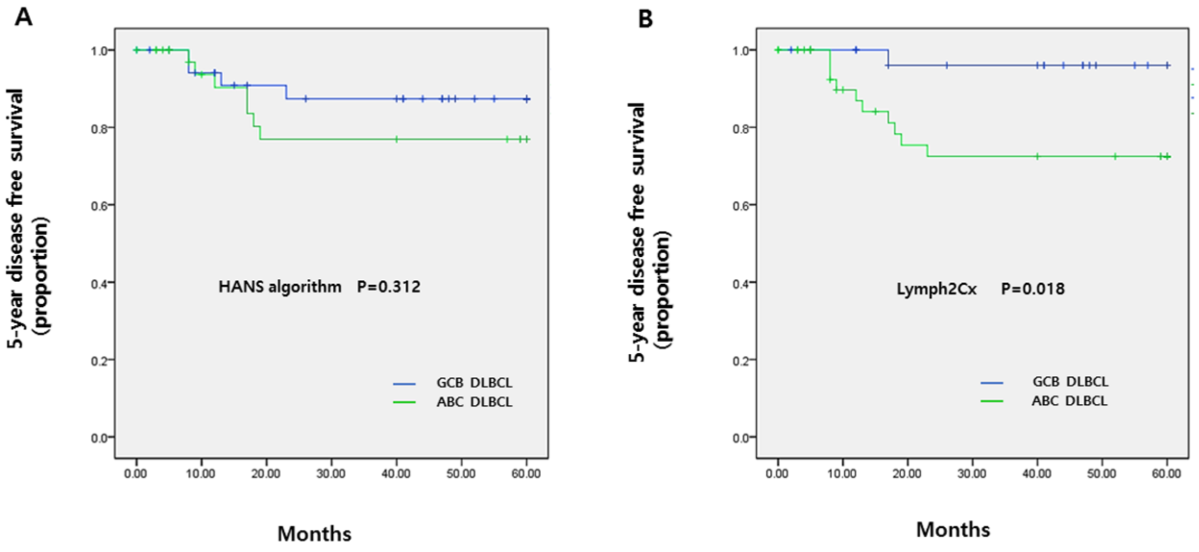

Supplementary Figure 2: Kaplan–Meier analyses of 5-year DFS in the patients with DLBCL types classified by the Hans algorithm A. or Lymph2Cx assay B. excluding unclassified groups by the Lymph2Cx assay. (A) COO assay by the Hans algorithm showed no difference in 5-year OS ($P = 0.312$) between the GCB and non-GCB types. (B) Patients with the Lymph2Cx-defined GCB had significantly better 5-year DFS outcomes than those with the ABC type (GCB vs. ABC; 96.6% vs. 79.2%, $P = 0.018$).
